# A cyclic di-GMP phosphodiesterase in the VSP-2 island of *Vibrio cholerae* is regulated by zinc and quorum sensing

**DOI:** 10.1128/mbio.02275-25

**Published:** 2025-09-24

**Authors:** Aathmaja Anandhi Rangarajan, Kiwon Ok, Marissa K. Malleck, Micah J. Ferrell, Thomas V. O'Halloran, Christopher M. Waters

**Affiliations:** 1 Department of Microbiology Genetics and Immunology, Michigan State University 3078, East Lansing, Michigan, USA; 2 Elemental Health Institute, Michigan State University 3078, East Lansing, Michigan, USA; 3 Department of Chemistry, Michigan State University 3078, East Lansing, Michigan, USA; The Ohio State University, Columbus, Ohio, USA

**Keywords:** zinc, cyclic di-GMP, *Vibrio*, *cholerae*, quorum sensing

## Abstract

The second messenger cyclic di-GMP (cdG) is indispensable for the regulation of biofilm formation, motility, and a variety of other important bacterial behaviors in the majority of bacteria. The human pathogen *Vibrio cholerae* has a diverse repertoire of diguanylate cyclase and phosphodiesterase (PDE) enzymes that control the intracellular cdG concentration depending on local environmental cues and its physiological state. Determining the transcriptional regulation of these enzymes and the respective environmental signals that control their activity is important to understand how and when *V. cholerae* switches between motile and sessile lifestyles in different environments. In some strains of the current *V. cholerae* seventh pandemic El Tor biotype, the horizontally acquired *Vibrio* seventh pandemic 2 (VSP-2) island encodes an uncharacterized PDE at the gene locus *vc0515*, which we named *zpdA* (zinc-inhibited phosphodiesterase A). We show, here, that *zpdA* transcription is repressed by Zur when Zn^2+^ is abundant, as well as by the quorum sensing regulator HapR when cells grow to high density. Furthermore, we find that the PDE activity of the purified ZpdA protein is inhibited by Zn^2+^ but is dependent on alternative divalent cations such as Mn^2+^, which we find are elevated in *V. cholerae* cells grown under zinc-limiting conditions. We conclude that ZpdA is an active metal-dependent PDE that is regulated by Zn^2+^ availability at both the level of transcriptional and post-translational leading to elevated cdG levels when Zn^2+^ is abundant. Our results demonstrate the important role of metal availability in modulating cdG signaling in bacteria.

**IMPORTANCE:**

*Vibrio cholerae* colonizes estuarine environments and human hosts, where it transitions between motile and sessile states using the second messenger cyclic di-GMP (cdG). cdG levels change in response to a variety of signals and are controlled by the activity of diguanylate cyclases and phosphodiesterase (PDEs), enzymes that make and degrade cdG. In this work, we show that Zn^2+^ and the cell density regulator HapR repress ZpdA, the PDE present in the *Vibrio* seventh pandemic 2 island, at the level of transcription, and that Zn^2+^ unexpectedly alters the PDE activity of ZpdA protein itself. Our study highlights the role of metal availability as an important signaling cue that controls *V. cholerae* biology.

## INTRODUCTION

*Vibrio cholerae* is a bacterial pathogen capable of causing cholera. One distinguishing feature of the El Tor biotypes, which are responsible for the seventh and current pandemic of *V. cholerae*, from the previous classical biotype is the acquisition of the *Vibrio* seventh pandemic 1 (VSP-1) and *Vibrio* seventh pandemic 2 (VSP-2) islands. These islands are hypothesized to have aided El Tor in displacing the classical biotype in both environmental and clinical reservoirs (1, 2). Although many of the gene functions in these genomic islands are unknown, some of the genes of VSP-1 and VSP-2 islands are involved in defense against phages and foreign DNA (3–6). In addition to infecting humans, *V. cholerae* is associated with various species in aquatic environments where it is challenged with diverse stressors. Thus, strategies to successfully switch between motile and sessile forms in these environments are also likely key to the emergence of El Tor (7).

The second messenger cyclic di-GMP (cdG) controls several important physiological processes, including, but not limited to, biofilm formation, motility, type two secretion, DNA repair, cell shape, and colonization (8–12). The intracellular cdG concentration is a major driver of the motile-sessile lifestyle switch, whereby high levels of cdG promote biofilm formation and low levels induce motility (10). Intracellular cdG concentrations are dictated by the regulation of GGDEF-containing diguanylate cyclases (DGCs), which synthesize cdG, and EAL/HD-GYP-containing phosphodiesterases (PDEs), which degrade it (8, 9). The first sequenced strain of *V. cholerae*, N16961, possesses 31 GGDEF-containing proteins, 10 that contain both an EAL and GGDEF, 12 that contain EAL, and 8 that contain HD-GYP domains, although such numbers can exhibit minor variation from strain to strain (8, 10). For example, one of the PDE-containing proteins, *vc0515*, which we rename in this work to *zpdA* for zinc-inhibited phosphodiesterase A, is encoded in the VSP-2 island in only some strains of El Tor, suggesting a horizontally acquired PDE can potentially impact cdG levels and thereby regulate biofilm formation and motility (13). *zpdA* (*vc0515*) lies downstream of transcriptional activator *verA* (*vc0513*) and *vc0514*, presumably in an operon, and all three genes (*vc0513-15*) are repressed by Zur in the presence of zinc. Under zinc-limiting conditions, or in the absence of Zur, *verA*, v*c0514*, and *zpdA* genes are derepressed, with VerA further enhancing its expression by binding to the *verA* promoter (13). ZpdA is a predicted PDE, but the contribution of ZpdA to intracellular cdG concentrations, biofilm formation, and motility, nor the environmental signals that regulate this protein, have not been studied.

Most cdG DGCs or PDEs possess one or more N-terminal sensory domains that are thought to assimilate and respond to specific signals (8, 14). However, only a few signals, such as nitric oxide, bile, spermidine, light, and oxygen, have been described to regulate specific DGC and PDE activity in *V. cholerae*, thereby modulating intracellular cdG (14, 15). Metals such as Zn^2+^, Fe^3+^, Mn^2+^, and Mg^2+^ are essential nutrients, which, unlike other organic enzymatic co-factors, can neither be synthesized nor destroyed. Thus, the intracellular concentrations of these nutrients are maintained in narrow ranges through tightly regulated homeostatic mechanisms under high and low metal conditions (16–18). Mn^2+^, Ca^2+^, and Fe^3+^ were shown to regulate the *in vitro* activity of VieA, *Vc*EAL, and VCA0681, respectively, in *V. cholerae* (19–21). Besides these *in vitro* studies, whether and how these metals impact cdG regulation in *V. cholerae* or, more broadly, in other bacterial species is not understood.

Here, we show that the *zpdA* PDE allows *V. cholerae* to regulate cdG levels in response to Zn^2+^ availability both at the level of transcriptional by Zur and at post-translation level by inhibiting the ZpdA PDE activity. We also demonstrate that *zpdA* is repressed by quorum sensing (QS). Within the highly variable VSP-2 region, the *zpdA* gene is highly conserved in several El Tor strains, underscoring the potential role of Zn^2+^ modulation of cdG in the environmental persistence of the seventh *V. cholerae* pandemic.

## RESULTS

### ZpdA is an active PDE encoded in the VSP-2 island

**Fig 1:**
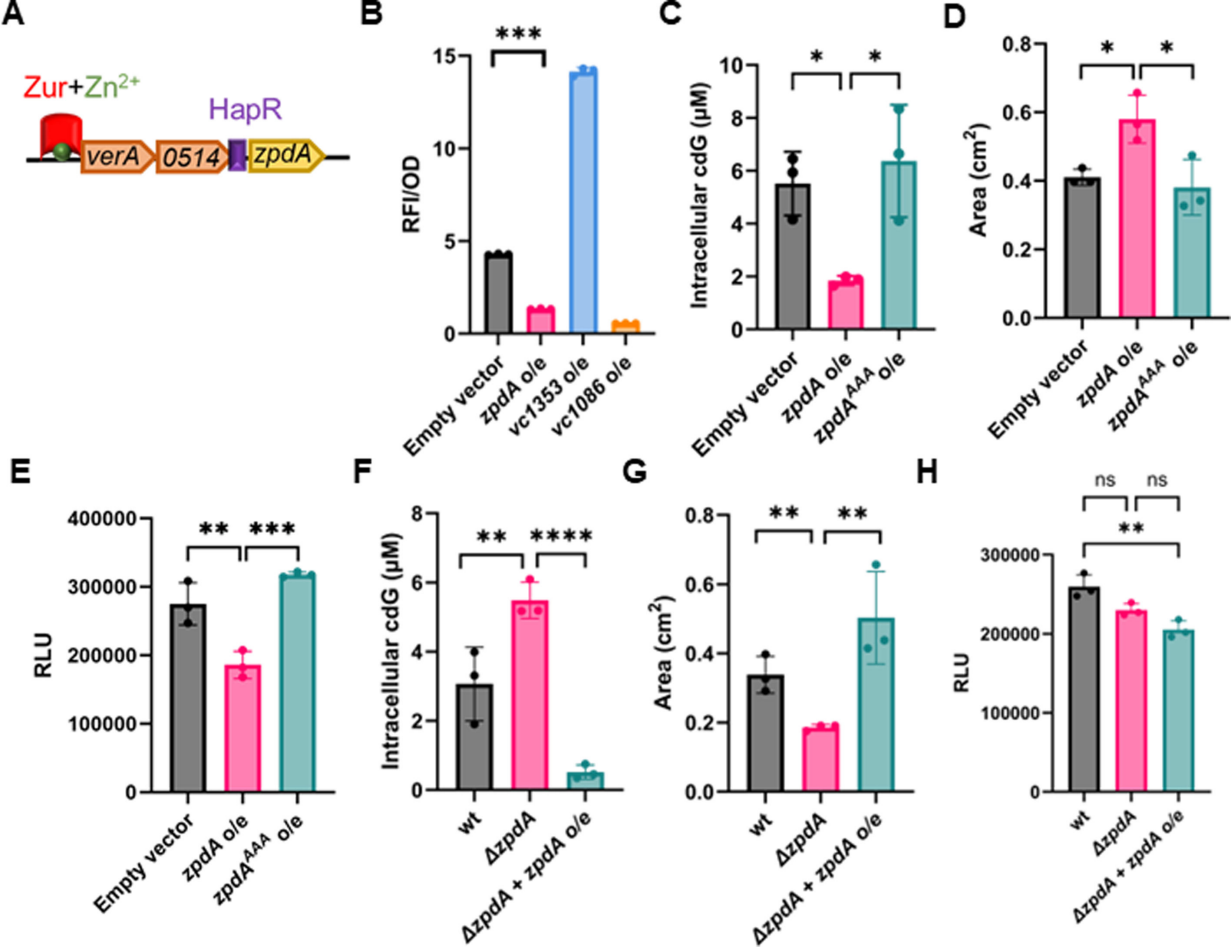
ZpdA is an active phosphodiesterase. (**A**) Genomic loci map of *verA*, *vc0514*, and *zpdA* genes. Zur repressor (red) forms a complex with divalent zinc ions (green) and binds to the *verA* promoter. Potential HapR binding site (purple) upstream of *zpdA* coding region is shown. (**B**) cdG binding RFP biosensor readout normalized to optical density at 600 nm (OD_600_) from overexpression of DGC (*vc1353*), PDE (*vc1086*), PDE (*zpdA*), and empty vector (pEVS141) with 1 mM IPTG. (**C**) Intracellular cdG levels in strains overexpressing *zpdA*, *zpdA^AAA^*, or empty vector (pEVS141) with 1 mM IPTG. (**D**) Quantification of motility by measuring the colony area in strains overexpressing *zpdA*, *zpdA^AAA^*, or empty vector (pEVS141) with 1 mM IPTG. (**E**) Quantification of biofilm expressed as relative luminescence units in strains overexpressing *zpdA*, *zpdA^AAA^*, or empty vector (pEVS141) with 1 mM IPTG. (**F**) Intracellular cdG levels in wild type, Δ*zpdA*, and Δ*zpdA* with overexpression of *zpdA*. (**G**) Quantification of motility by measuring the colony area in strains in wild type, Δ*zpdA*, and Δ*zpdA* with overexpression of *zpdA*. (**H**) Quantification of biofilm expressed as relative luminescence units in wild type, Δ*zpdA*, and Δ*zpdA* with overexpression of *zpdA*. N16961 strain background was used for all of these assays. The mean and standard deviation of three biological replicates are indicated. Statistical significance was determined by one-way analysis of variance (ANOVA) and *post hoc* Tukey test (*, *P* < 0.05; ***, *P* < 0.005; ****, *P* < 0.0005; ns, not significant).

The *zpdA* (*vc0515*) gene encodes a predicted EAL PDE. This gene is located in the *vc0513-15* operon of the VSP-2 island of some El Tor strains, including N16961, and this operon was previously demonstrated to be repressed by the Zn^2+^-responsive repressor Zur (13) (Fig. 1A). Unlike most PDEs, the EAL domain of ZpdA is encoded on the N-terminus of the protein, while the C-terminus encodes an HDOD domain, which has been shown to be regulated by metals (22, 23). We therefore sought to determine the impact of ZpdA, and its modulation by Zur and Zn^2+^, on intracellular cdG and associated phenotypes in *V. cholerae* strain N16961.

To determine if ZpdA is an active PDE, we overexpressed *zpdA* along with the cdG biosensor (pRP0122-*Pbe_amcyan_Bc3-4_turborfp*) in minimal media. This biosensor contains three cdG-responsive riboswitches that control the expression of *turboRFP* such that increased intracellular cdG leads to increased RFP (24). *zpdA* overexpression decreased RFP expression, similar to the PDE *vc1086*, which was previously shown to be active in these conditions, indicating a decrease in cdG (25). In contrast, expression of the DGC *vc1353* increased RFP levels, reflecting increased cdG (Fig. 1B). ZpdA possesses the amino acids “ELL” in its active site, and indeed overexpression of the active site mutant (ELL-AAA) of *zpdA*, which we name *zpdA^AAA^*, followed by direct quantification of intracellular cdG using liquid chromatography-tandem mass spectrometry (LC-MS/MS), indicated that wild-type (WT) *zpdA* reduced cdG while these mutations rendered ZpdA inactive (Fig. 1C). Consistent with its impact on intracellular cdG, overexpression of *zpdA* increased motility and decreased biofilm formation when compared to the empty vector or the *zpdA^AAA^* (Fig. 1D and E).

To determine whether *zpdA* impacted cdG at native expression levels, we measured the intracellular cdG levels in the WT strain and an isogenic Δ*zpdA* mutant in M9 minimal media. The Δ*zpdA* mutation exhibited higher intracellular cdG compared to the WT strain, which could be offset by expression of *zpdA* from a plasmid (Fig. 1F). The elevated intracellular levels of cdG in the Δ*zpdA* mutant led to decreased motility (Fig. 1G). However, the Δ*zpdA* mutant did not significantly impact biofilm formation relative to the wild-type strain (Fig. 1H). Importantly, measuring cdG, motility, and biofilms requires different growth conditions that might impact the activity or expression of ZpdA. Moreover, given that *V. cholerae* encodes dozens of PDEs and cdG concentrations can be modulated via negative feedback loops, it is common not to observe a significant phenotypic difference when only one is deleted (25). The summation of these results indicates that *zpdA* is an active PDE in the conditions examined here that decreases intracellular cdG, impacting downstream cdG-regulated phenotypes.

### Zn^2+^ inhibits the expression of *zpdA*

Transcription of the *zpdA* gene is repressed by the Zur repressor in the presence of elevated Zn^2+^ availability through the upstream *verA* (*vc0513*) promoter (13) (Fig. 1A). Deletion of the *zur* gene did not significantly impact intracellular cdG and motility while leading to decreased biofilm formation (Fig. S1A through C). Decreased biofilm formation in the *zur* mutant could be due to the downregulation of several *rbm* and *vps* operon genes involved in biofilm formation, as previously demonstrated in a transcriptomic analysis (13).

**Fig 2:**
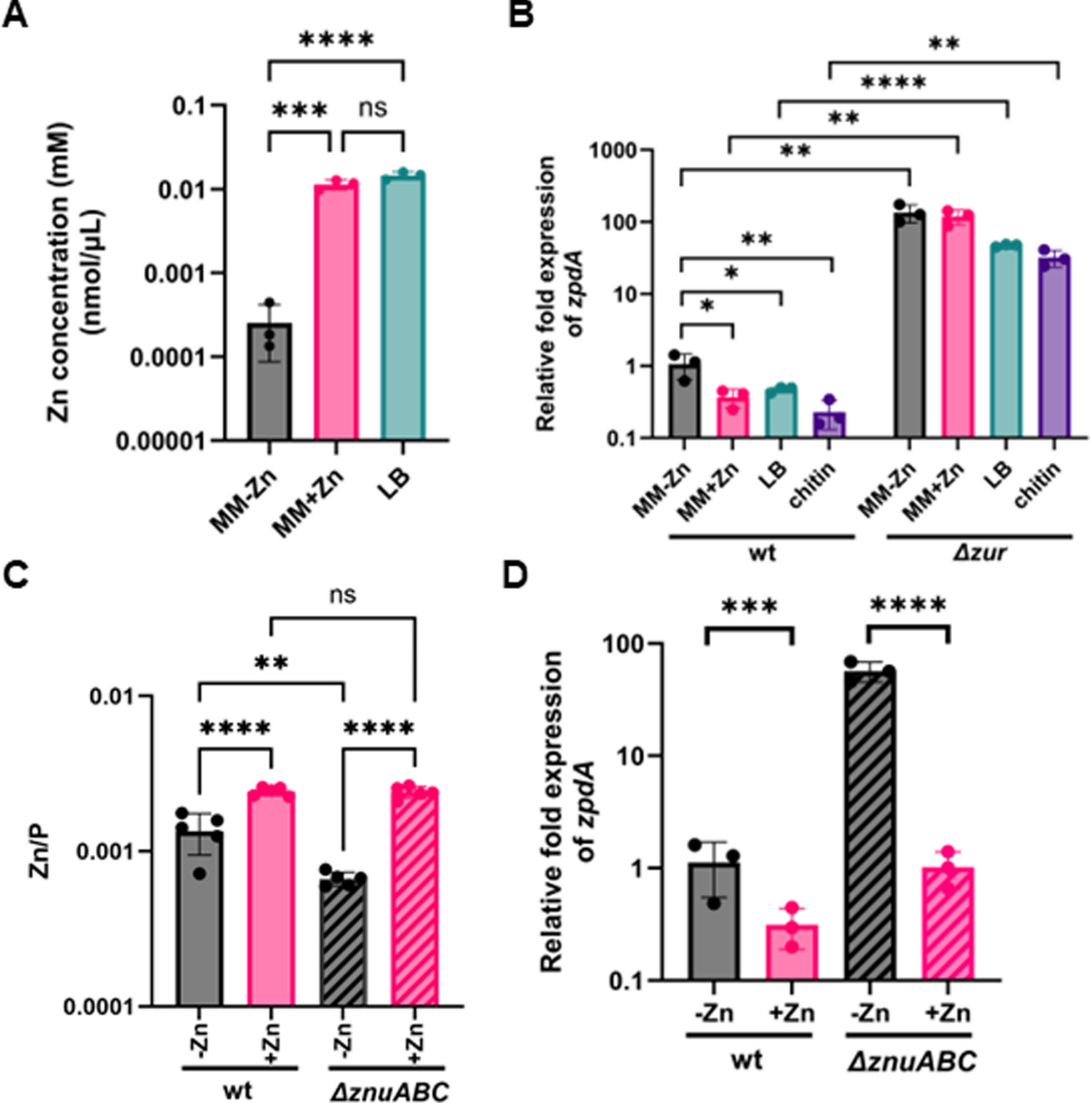
Zur in the presence of Zn^2+^ represses expression of the *zpdA* phosphodiesterase. (**A**) ICP-MS quantification of zinc content in minimal media, minimal media with zinc sulfate (10 µM), and LB media. The average of three independent replicates is indicated. (**B**) Relative expression of *zpdA* determined by qPCR in N16961 wild type and Δ*zur* in minimal media, minimal media with zinc, chitin, and LB. (**C**) ICP-MS quantification of intracellular zinc normalized to phosphorus in N16961 wild type and Δ*znuABC* mutant in minimal media with and without zinc supplementation (10 μM ZnSO_4_). (**D**) Relative expression of *zpdA* determined by qPCR in N16961 wild type and Δ*znuABC* in minimal media and minimal media plus zinc. The mean and standard deviation of three biological replicates are indicated. Statistical significance was determined by one-way ANOVA and *post hoc* Tukey test (*, *P* < 0.05; ***, *P* < 0.005; ****, *P* < 0.0005; ns, not significant).

Given that the interpretation of the Δ*zur* mutant is challenging due to pleiotropic cdG-independent regulation (13), we analyzed the impact of Zn^2+^ on *zpdA* expression. First, we sought to understand how zinc levels changed in different growth conditions and after adding extracellular Zn^2+^. We measured the level of zinc in different media powders and final growth media using inductively coupled plasma mass spectrometry (ICP-MS). As a control, we added 10 µM of Zn^2+^ to minimal media and measured it alongside other media. Minimal media, minimal media with zinc, and lysogeny broth (LB) media contained 0.03 µM, 11 µM, and 15 µM of zinc, respectively (Fig. 2A). *Vc* colonizes crustaceans as biofilms and utilizes chitin as a carbon source; hence, we also measured the level of zinc in chitin (26, 27). In the laboratory, *Vc* is grown with chitin flakes in Instant Ocean Water medium resembling the ocean water, as described in Materials and Methods. Therefore, we analyzed the zinc content of chitin flakes from shrimp and crab alongside instant ocean salts. Shrimp chitin (0.0014 ng/µg) and crab chitin (0.06 ng/µg) had significantly higher zinc content than instant ocean salts alone (0.00013 ng/µg) (Fig. S2).

With knowledge of zinc concentrations in these media, we next determined the relative expression of *zpdA* in different media in the WT strain and Δ*zur* mutant using quantitaive reverse transcriptase PCR (qRT-PCR). In the WT strain, the relative expression of *zpdA* was decreased 2–5-fold in LB, crab chitin, and minimal media with zinc supplementation (10 µM) when compared to minimal media alone (Fig. 2B). We observed a more dramatic effect in the Δ*zur* strain, where expression of *zpdA* was 126-fold higher in minimal media and was non-responsive to zinc addition, being 320-fold higher. We also observed 100-fold and 138-fold higher expression of *zpdA* in the Δ*zur* strain in zinc-rich LB and chitin media, respectively (Fig. 2B). This result clearly shows that Zur is an active repressor in all media examined, which is likely due to the sub-femtomolar affinity of Zur for zinc, leading to repression even at low zinc concentrations (17).

A previous study showed derepression of the upstream *verA* promoter using the zinc importer Δ*znuABC* mutant (13). We therefore reasoned that this mutant could be used to assess the effects of zinc on *zpdA* repression, given that it may have lower intracellular concentrations of zinc. To confirm this, we quantified the intracellular concentrations of zinc and normalized them to phosphorus levels in each sample, which is an established internal standard for cell biomass using ICP-MS (28). Normalized zinc levels were twofold lower in the Δ*znuABC* mutant relative to wild type. Adding 10 µM ZnSO_4_ to minimal media increased the intracellular zinc levels in both wild-type and Δ*znuABC* strains (Fig. 2C). High zinc concentrations in the Δ*znuABC* mutant upon zinc addition are likely due to secondary zinc transporters (29). In line with the intracellular zinc levels, the relative expression of the *zpdA* gene was 57-fold higher in the Δ*znuABC* mutant when compared to the WT, and the addition of zinc strongly repressed *zpdA* expression (Fig. 2D). These results demonstrate that changes in the intracellular concentration of zinc modulate Zur repression of *zpdA*, but normally, laboratory growth conditions have sufficient intracellular Zn^2+^ for it to function as a Zur corepressor.

**Fig 3:**
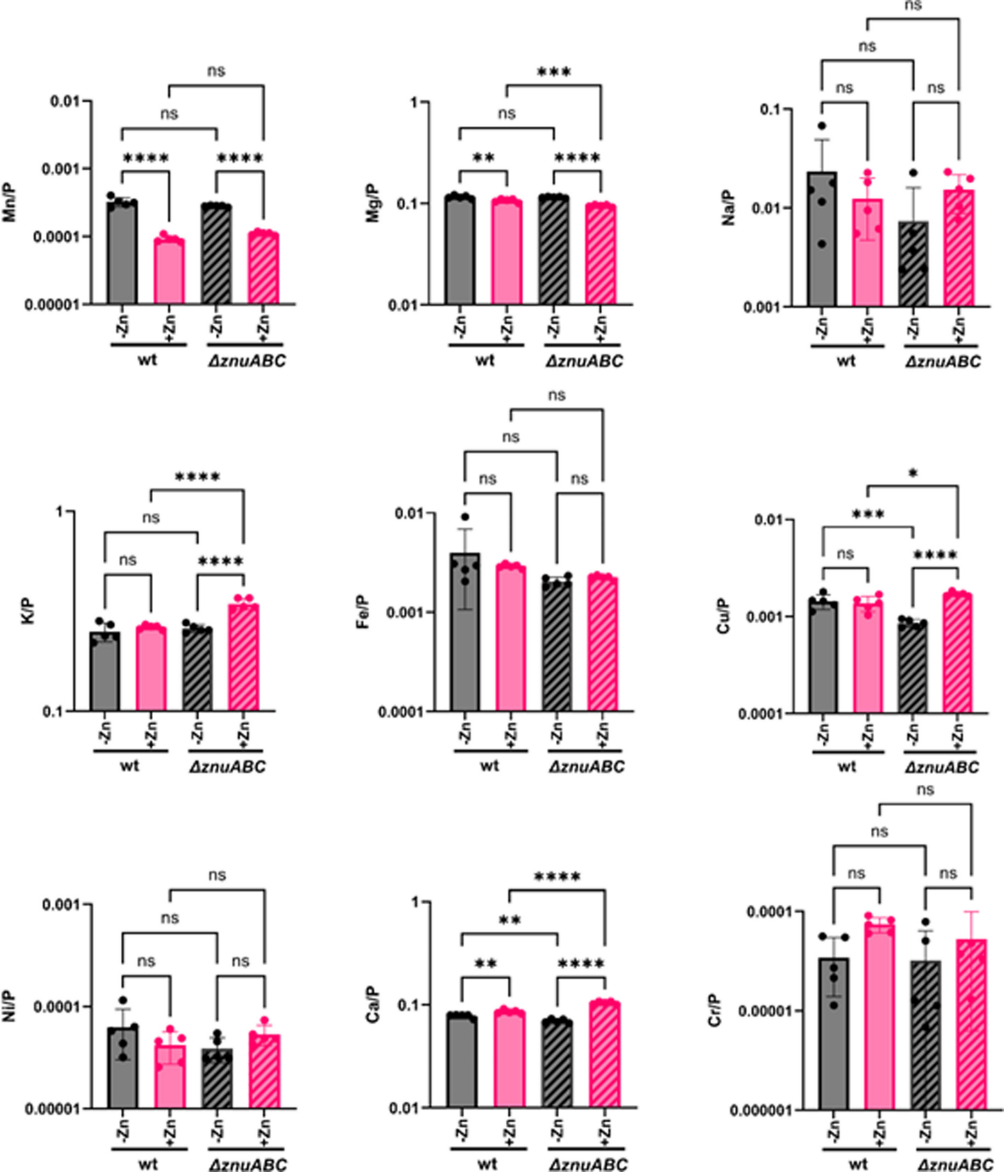
Quantification of metals in high and low zinc. Intracellular metal concentrations of Mn, Mg, Na, K, Fe, Ni, Ca, Cu, and Cr (note: different charge forms cannot be discriminated by ICP-MS) contents normalized to phosphorus atoms in N16961 wild type and the Δ*znuABC* mutant grown in minimal media and minimal media supplemented with 10 µM ZnSO_4_ determined by ICP-MS. Mean and standard deviation of three biological replicates are indicated. Statistical significance was determined by one-way ANOVA and *post hoc* Tukey test (*, *P* < 0.05; ***, *P* < 0.005; ****, *P* < 0.0005; ns, not significant).

To assess the impact of ZnuABC on the import of other metals, we also measured other metal ions in the WT and Δ*znuABC* strains with and without additional zinc using ICP-MS. There was no significant difference in Mn, Mg, Na, K, Fe, Ni, and Cr levels (Fig. 3), but cellular Cu content is significantly lower in the Δ*znuABC* mutant relative to WT, and the magnitude of this difference is similar to that observed for Zn (Fig. 2C and 3). This is consistent with well-documented crosstalk between Cu and Zn homeostasis networks in both gram-negative and gram-positive organisms (30–32); however, the specific mechanisms have yet to be resolved. The largest difference in metal content was observed for cells grown in minimal media supplemented with Zn. Under these conditions, Mn levels were 3.5-fold higher when cells were grown without zinc supplementation. There were several other statistically significant differences in metal concentrations upon zinc addition to the WT strain or when comparing the WT to the Δ*znuABC* mutant, but given that these differences are less than twofold, it is unclear if they are biologically meaningful. These results indicate that this elevated cellular Mn acquisition by *V. cholerae* is not mediated by the *znuABC* transport system (Fig. 3) and open the question as to why cells acquire so much more manganese under zinc limitation conditions.

### Repression of *zpdA* by the QS regulator HapR

**Fig 4:**
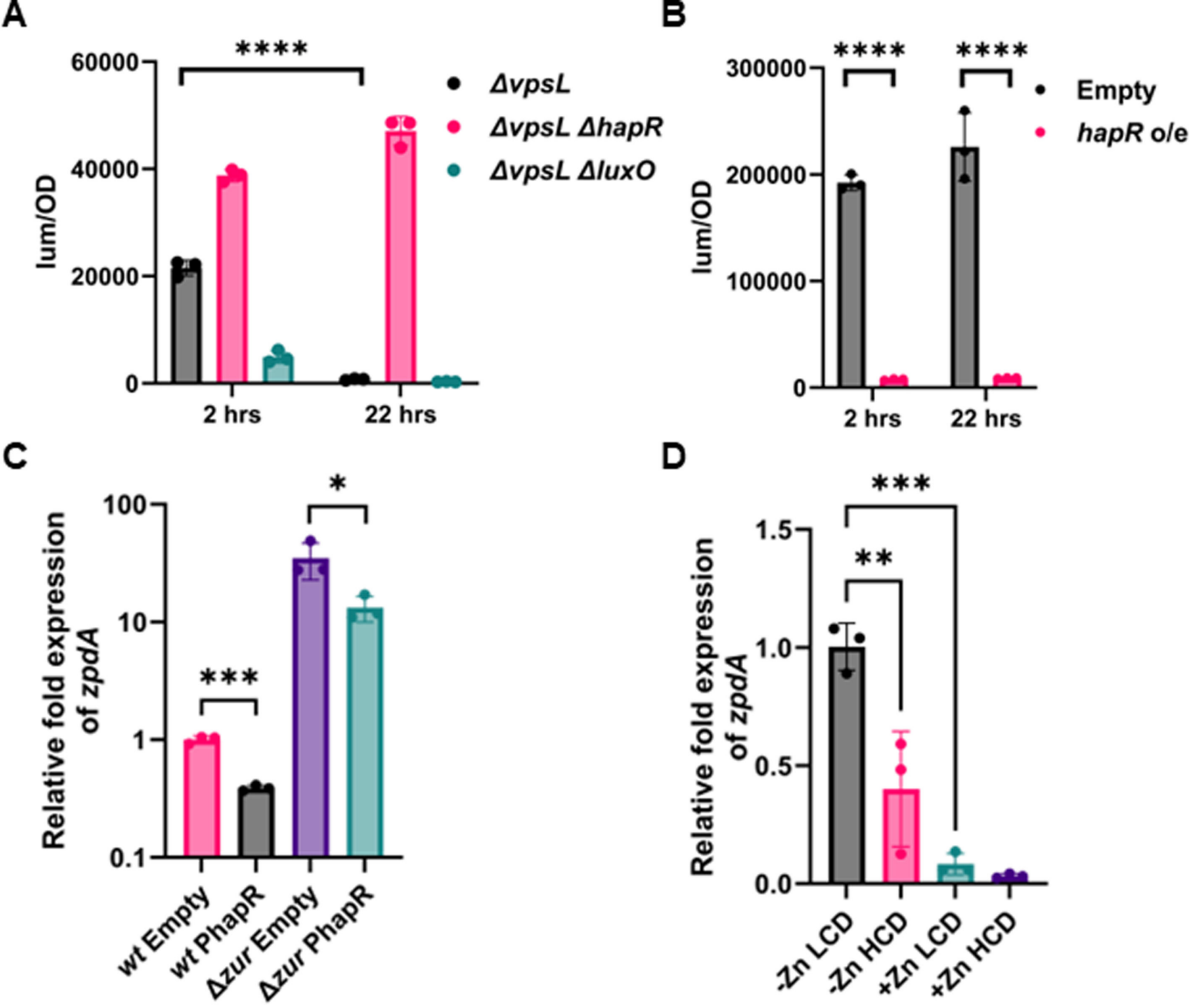
HapR represses the *zpdA* promoter. (**A**) Luminescence measured from the expression of *P_zpdA_-lux* normalized to OD_600_ in Δ*vpsL*, Δ*vpsL* Δ*luxO*, and Δ*vpsL* Δ*hapR* strains of C6706 at 2 hours and 22 hours. (**B**) Luminescence measured from the overexpression of HapR and empty vector normalized to OD_600_ in Δ*vpsL* strain of N16961. (**C**) Relative expression of *zpdA* determined by qPCR in wild type and *Δzur* strains of N16961 with overexpression of HapR or empty vector. (**D**) Relative expression of *zpdA* in E7646 strain at low cell density (LCD) (OD_600_: 0.1) and high cell density (HCD) (OD_600_: 1.5–2) in minimal media with and without Zn^2+^. Mean and standard deviation of three biological replicates are indicated. Statistical significance was determined by one-way ANOVA and *post hoc* Tukey test (*, *P* < 0.05; ***, *P* < 0.005; ****, *P* < 0.0005).

*zpdA* is part of an operon of genes containing *verA*, *vc0514*, and *zpdA* (Fig. 1A), and it is thought to be expressed as a single gene transcript via the *verA* promoter (13). However, bioinformatic analysis predicted a HapR binding site directly upstream of the *zpdA* coding region (33). HapR is the master QS regulator in *V. cholerae* that is expressed when cells transition to high cell density (HCD), regulating hundreds of genes, including those coding for DGCs and PDEs (33, 34). To determine if *zpdA* is directly regulated by HapR, we generated a plasmid-encoded transcriptional fusion of the 100 bp upstream of *zpdA* containing the putative HapR binding site to *luxCDABE*. Since *V. cholerae* strain N16961 encodes a non-functional *hapR*, which has been observed for many El Tor strains (35, 36), we analyzed expression of P*_zpdA_-lux* at the low and high cell density states using the QS-competent *V. cholerae* strain C6706. We also examined a Δ*luxO* mutant (locking the cells at high cell density) and a Δ*hapR* mutant (locking the cells at low cell density [LCD]). Each of these strains also encoded a deletion of *vpsL* to abolish biofilm formation, enabling accurate readout of the transcriptional reporter. P*_zpdA_-lux* expression was higher in the QS WT strain (Δ*vpsL*) at low cell density when compared to the high cell density condition where HapR is expressed (Fig. 4A). Similarly, the expression was constitutively high in the Δ*hapR* Δ*vpsL* mutant and repressed in Δ*luxO* Δ*vpsL* where HapR is constitutively expressed (Fig. 4A). Overexpression of HapR in strain N16961 had a similar effect in driving P*_zpdA_-*lux repression (Fig. 4B). These results, combined with the predicted HapR binding site, suggest that HapR can bind to the *zpdA* upstream region to repress its expression.

Our results demonstrate that both Zur and HapR repress expression of *zpdA*. To disentangle the impact of each of these regulators, we tested the relative expression of *zpdA* using qRT-PCR upon overexpression of HapR in WT N16961 and the Δ*zur* mutant. HapR overexpression reduced the expression of *zpdA* 2.6-fold in WT and Δ*zur* mutant (Fig. 4C). However, deletion of *zur* alone increased expression of *zpdA* 35-fold, suggesting that in strain N16961 under the conditions tested here, Zur had a larger impact on *zpdA* expression compared with HapR.

We next tested the relative impact of Zn^2+^ and HapR on the expression of *zpdA* under native conditions without HapR overexpression. As N16961 does not encode a functional *hapR* and C6706 does not encode the *zpdA* gene, we performed this experiment in strain E7646, which encodes both a functional *hapR* and the *verA-zpdA* operon in the VSP-2 island. We performed this experiment in M9 minimal media with and without Zn^2+^ at low and high cell densities. The addition of Zn^2+^ to this strain significantly decreased expression of *zpdA*, but QS regulation was evident in both conditions as the relative expression of *zpdA* was reduced 2.5-fold and 2.6-fold between low and high cell density in low and high Zn^2+^ (Fig. 4D). Similar effects of QS in strain E7646 grown on chitin flakes and in LB medium were also observed (Fig. S3A). These results demonstrate that both Zur and HapR regulate the expression of *zpdA*, with Zur playing the dominant role while HapR modulates expression in the conditions tested.

### Zn^2+^ inhibits the PDE activity of ZpdA

cdG signaling allows bacteria to sense environmental changes to regulate biofilm formation and motility. As demonstrated above, Zn^2+^ represses *zpdA* transcription; however, given that the already expressed ZpdA enzyme would remain in cell for some time, such a transcriptional response would exhibit significant phenotypic lag before cdG levels were impacted. We thus considered whether Zn^2+^ impacted ZpdA in a post-transcriptional manner, leading to a more rapid response of cdG levels to changing Zn^2+^ concentrations.

**Fig 5:**
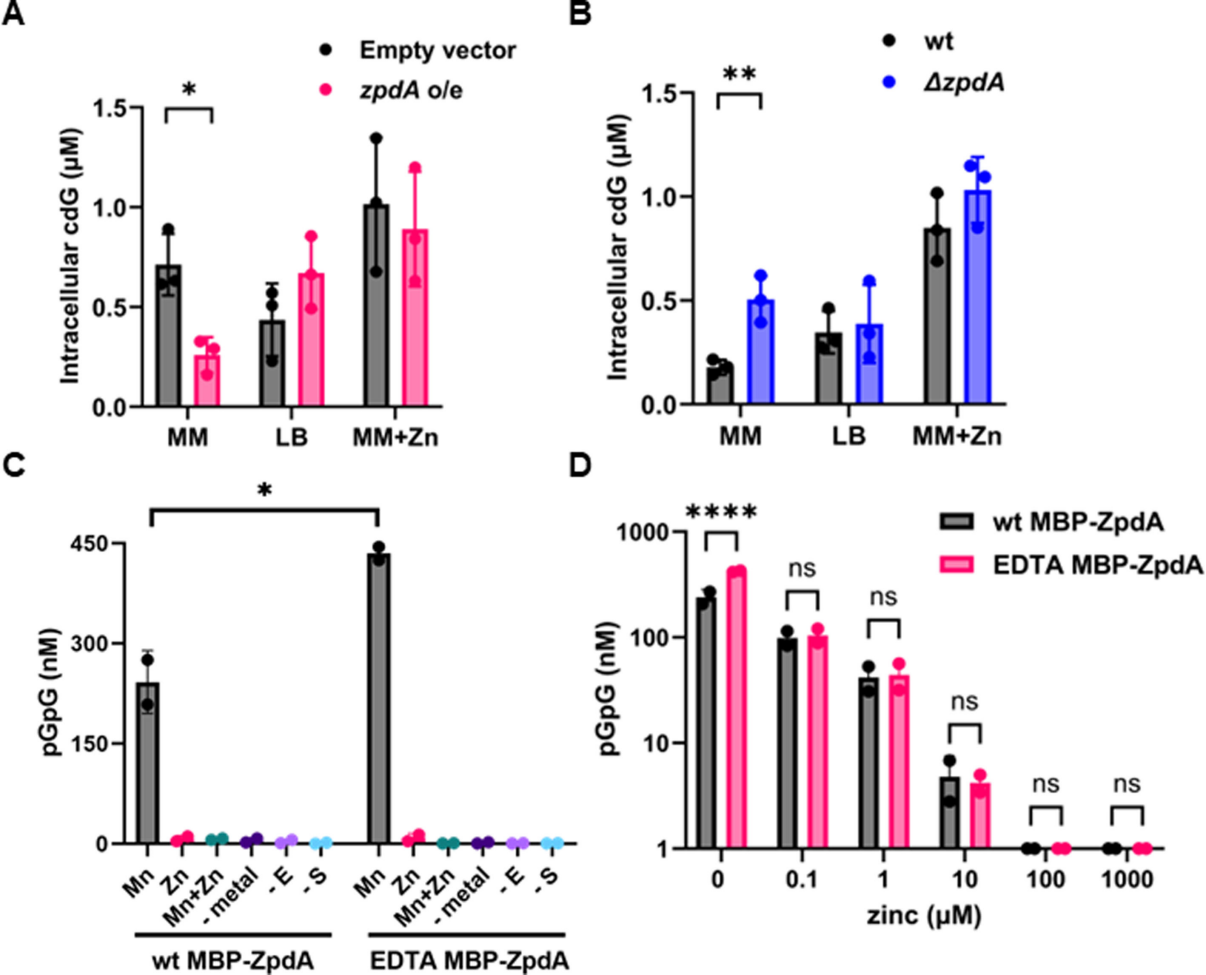
Zn^2+^ inhibits the protein activity of ZpdA. (**A**) Intracellular cdG levels with overexpression of *zpdA* and empty vector in minimal media and LB in N16961 strain. (**B**) Intracellular cdG levels of wild type and Δ*zpdA* in minimal media and LB in N16961 strain. (**C**) Concentration of pGpG measured by LC-MS/MS in wild type and EDTA-treated protein with cdG substrate in the presence of 1 mM MnCl_2_, 1 mM ZnCl_2_, and 1 mM MnCl_2_ + ZnCl_2_ along with no metal, no substrate, and no enzyme controls. (**D**) Concentration of pGpG produced from cdG measured by LC-MS/MS in samples of the as-purified protein and EDTA-treated protein with 1 mM Mn^2+^ and different concentrations of Zn^2+^ (0, 0.1, 1, 10, 100, and 1,000 µM). Mean and standard deviation of three replicates are indicated. Statistical significance was determined by *t*-test (*, *P* < 0.05; ***, *P* < 0.005; ****, *P* < 0.0005; ns, not significant).

In support of this idea, when we overexpressed *zpdA* from a plasmid in strain N16961, we found that ZpdA was active and decreased cdG levels in M9 minimal media but not in LB or minimal media supplemented with Zn^2+^ as measured by direct quantification of cdG (Fig. 5A) or the cdG biosensor (Fig. S3B). Moreover, Δ*zpdA* only exhibited a significant difference in the intracellular levels of cdG compared to the WT strain when grown in minimal media but not in LB or Zn^2+^-supplemented minimal media (Fig. 5B). As LB medium is rich in Zn^2+^, these results suggested that Zn^2+^ may directly inhibit the PDE activity of ZpdA.

To test whether Zn^2+^ inhibits the activity of ZpdA, we purified an MBP-ZpdA fusion protein in the presence and absence of EDTA. We used EDTA treatment to remove any inhibitory metals that might bind to ZpdA. We tested the PDE activity of EDTA-treated and untreated MBP-ZpdA using cdG as substrate by measuring the accumulation of 5′-pGpG-3′ using mass LC-MS/MS. Interestingly, without any metal addition, ZpdA was inactive (Fig. 5C).

While the mechanisms of manganese uptake are not yet clear, the increased Mn content under zinc-limiting conditions led us to test for a biochemical role for this divalent metal. We found that MBP-ZpdA exhibited PDE activity only when 1 mM MnCl_2_ or, to a lesser extent, 1 mM MgCl_2_ was added to the reaction (Fig. 5C; Fig. S3C). Addition of 1 mM ZnCl_2_, FeSO_4_, or CaCl_2_ did not stimulate PDE activity, nor did addition of a combination of MnCl_2_ and ZnCl_2_ (Fig. 5C; Fig. S3C). This result led us to test whether Zn^2+^ was inhibiting PDE activity by conducting a dose-response assay. In the presence of 1 mM MnCl_2_, we find that the PDE activity of MBP-ZpdA was decreased in a dose-dependent manner by increasing concentrations of Zn^2+^ (Fig. 5D). Additionally, we find that the Mn^2+^-induced PDE activity was higher for MBP-ZpdA samples that were purified in the presence of EDTA vs the absence of EDTA (Fig. 5C and D; Fig. S3D). The most likely explanation is that EDTA removed any contaminating Zn^2+^, allowing for higher levels of Mn^2+^ loading and thus leading to higher levels of enzyme activity. Taken together, these experiments demonstrate that Zn^2+^ inhibits the PDE activity of ZpdA protein, whereas Mn^2+^ and, to a lesser extent, magnesium activate it. This finding aligns with our *in vivo* experiments, which suggest that ZpdA remains inactive at high Zn^2+^ concentrations.

## DISCUSSION

Although the presence of the VSP-1 and VSP-2 islands is a hallmark of the current *V. cholerae* El Tor biotype, multiple variants of VSP-2 have been identified (2, 37). Our analysis and others have revealed that the *vc0513-15* genes in the VSP-2 island genes are highly conserved among several El Tor isolates tested (Fig. S4) (37, 38). For example, a recent isolate responsible for a major outbreak in Bangladesh (BD-1.2) lacks genes *vc0495-vc0512* in the VSP-2 island but encodes *vc0513-15* (Fig. S4) (39, 40). Conservation of these genes in most El Tor isolates underlines their evolutionary significance and potential role in environmental persistence.

**Fig 6:**
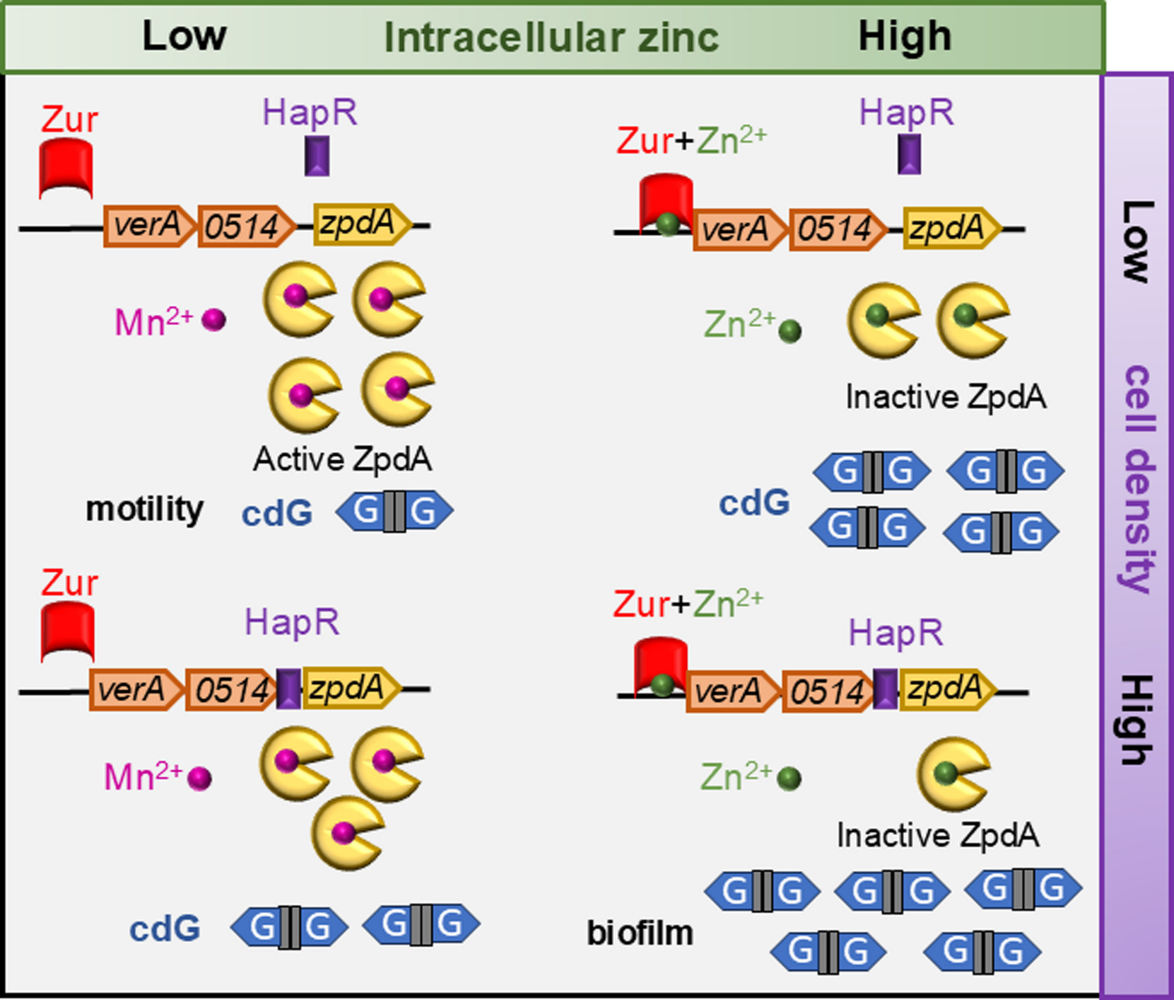
Zn^2+^ and quorum sensing regulate ZpdA. Under very low concentrations of Zn^2+^ and at low cell density, repression by Zur and HapR is relieved, resulting in high ZpdA active protein favoring a motile phenotype. At higher Zn^2+^ concentrations, Zur represses the upstream promoter; however, at low cell density, *zpdA* is expressed and the protein could be active or inactive depending on the intracellular Zn^2+^ levels. At high Zn^2+^ concentrations and at high cell density, Zur and HapR repress the expression of the *zpdA* gene, and ZpdA protein will be inactive due to the presence of high Zn^2+^, potentially favoring biofilm formation.

The QS regulator HapR has been shown to decrease global cdG levels by transcriptional regulation of DGCs and PDEs (34). This study was performed in strain C6706, which lacks *vc0513-15* (Fig. S4), and thus, the impact of *zpdA* regulation by QS was not assessed. Here, we demonstrate that HapR represses the *zpdA* promoter, although the magnitude of HapR repression on the expression of the *zpdA* gene is minimal when compared to Zur repression from the upstream *vc0513* promoter (Fig. 4C). Although Zur tightly binds to the upstream *verA* promoter and represses *zpdA*, LCD might allow partial de-repression of *zpdA*. N16961 strain with non-functional HapR, grown in minimal media, results in active ZpdA, albeit strongly repressed by Zur (Fig. 1F). Thus, QS provides additional regulation of *zpdA*, beyond Zur-mediated repression. The model described here is applicable to strain E7946 and/or other El Tor strains which have functional *hapR* and *zpdA* genes (Fig. 6). It is also known that several El Tor strains have null mutations in the *hapR* gene, resulting in elevated intracellular cdG (35, 36). Hence, the acquisition of *zpdA* in the VSP-2 island might reduce cdG levels in these strains, thereby enabling cdG homeostasis to effectively switch between motile and sessile phenotypes.

Environmental signals that regulate cdG levels are poorly understood. Bile acids, temperature, spermidine, oxygen, and cell density have been shown to regulate cdG levels (14). Many of the HD-GYP and EAL domains containing PDEs were shown to be bound by metals to regulate their activities *in vitro* (19). In *V. cholerae*, VC0681 binds to Fe^2+^ and Ca^2+^, VieA binds to Mn^2+^, and VC0395 binds to Ca^2+^ to stimulate their activity (20, 21, 41). Heme and Zn^2+^ were shown to inhibit the activity of the CdpA and VieA PDEs, respectively, in *V. cholerae* (20, 42). Four papers have demonstrated metal-mediated regulation of cdG *in vivo*. Mn^2+^ and Fe^2+^ activate and inhibit the PDE RbdA and DGC ImcA, respectively, resulting in reduced cdG levels in *Pseudomonas aeruginosa* (43, 44). Zn^2+^ inhibits the DGC DgcZ in *E. coli (*45) while Ca^2+^ activates the DGC CasA to increase cdG levels and biofilm formation in *Vibrio fischeri* (46). While a few *in vitro* studies have suggested that Zn^2+^ can inhibit PDE activity, our study is the first to establish Zn^2+^ as a bona fide *in vivo* signal modulating cdG via a PDE (Fig. 6) (20, 47). Importantly, we observed that physiologically relevant concentrations of Zn^2+^ impacted *zpdA* expression and activity and quantified metal levels in both environmental contexts, such as chitin, and within bacterial cells. These results provide new insight into how metal availability can directly influence cdG signaling and associated phenotypes.

We show ZpdA is active only in the presence of Mn^2+^ and to a lesser extent Mg^2+^ *in vitro* and that Zn^2+^ addition inhibits this activity (Fig. 5C; Fig. S3C). Mn^2+^ or Mg^2+^ binding to EAL domains was proposed to bridge a water molecule, forming a hydroxide ion, causing nucleophilic attack on the phosphorus atom of cdG, enabling the formation of pGpG (48, 49). The presence of Zn^2+^ could inhibit this nucleophilic attack and hence the PDE activity. The conserved metal binding residues that bind to Mn^2+^ or Mg^2+^ present in other EAL PDEs (48, 49) are also present, as evident in the AlphaFold model of ZpdA; hence, it is possible that ZpdA could modulate its activity via binding Mn^2+^ or Mg^2+^ (Fig. S5). Further studies are required to understand how Zn^2+^ might bind and/or inhibit ZpdA. In *V. cholerae*, two other PDEs, CdgJ (VC0137) and VC1851, and two PDEs from *Geobacter* and *Bacillus*, share similar domain structures to ZpdA, with N-terminal EAL domains and C-terminal HDOD domains (Fig. S6). Like ZpdA, these EAL enzymes may also modulate their PDE activity through Mn^2+^ and Zn^2+^, in response to environmental cues: we are currently exploring these possibilities (Fig. 6).

*Vibrio cholerae* likely encounters a variety of environments that have variable Zn^2+^ availability. For example, our results show that chitin contains significantly higher concentrations of Zn^2+^ when compared to ocean salts, and *V. cholerae* is highly proficient in forming biofilms in the presence of chitin (Fig. S2) (26, 50). The human host induces nutritional immunity by sequestering essential metals such as Zn^2+^ and Fe^3+^(16–18, 51, 52). Possibly, in environments with high Zn^2+^ concentrations, such as chitin, *V. cholerae* forms biofilms through the repression of ZpdA and other PDEs. In contrast, during human infections or in estuarine environments, *V. cholerae* may encounter low Zn^2+^, which triggers switching to an enhanced motility state. The gene upstream of *zpdA*, *vc0514*, might also enable motility as it was predicted to contain the histidine kinases, adenylyl cyclases, methyl-accepting proteins, and phosphatases (HAMP) domain along with a methyl-accepting chemotaxis domain. Previous findings have shown *verA* and *aerB* to be involved in aerotaxis (13). Hence, the presence of this operon in the VSP-2 island might be important for switching between motile and sessile phenotype by expression of *verA*, *vc0514*, and *zpdA* in low and high Zn^2+^ environments (Fig. 6). The Zn^2+^-responsive transcriptional control of this operon, modulated by QS, coupled with a Zn^2+^-responsive post-translational control of cdG levels, could be critical for environmental persistence. This behavior is reminiscent of uropathogenic *Escherichia coli*, where changes in Zn^2+^ availability provide cues that allow the organisms to persist and adapt to growth in diverse ecological niches (53). Of note, *zpdA* was not identified in a Tn-Seq analysis of genes important for virulence (54), and our examination of publicly available RNA-Seq transcriptomic data did not reveal any studies that showed differential regulation of *zpdA*. Our finding that *V. cholerae* imports up to 3.5-fold more Mn when switched from zinc-replete to zinc-limiting growth conditions, taken together with the finding that addition of Mn^2+^ stimulates PDE activity of purified ZpdA, suggests both Zn^2+^ and Mn^2+^ physiology influence the ability of *V. cholerae* to rapidly switch between motile to sessile states in response to a wide range of environmental stresses.

## MATERIALS AND METHODS

### Media and growth conditions

All bacterial strains and plasmids used in this study are listed in Table S1. *V. cholerae* strains used in this study were derived from N16961 unless otherwise indicated as E7946, C6706 Str2. *V. cholerae* strains were grown in LB, minimal medium (1× M9 minimal salts, 2 mM MgSO_4_, 0.1 mM CaCl_2_, 0.4% glucose), or chitin at 35°C with shaking. Chitin slurry is made as described previously with 8 g of crab chitin powder (Sigma-Aldrich) mixed in 150 mL of 0.5× Instant Ocean Water (7 g of instant ocean sea salts [Aquarium Systems, Inc.]/liter of water) (55). Minimal medium was prepared with ultrapure Milli-Q water to minimize metal contamination. ZnSO_4_ was used at a concentration of 10 µM. Cultures were supplemented with appropriate antibiotics when needed. Antibiotics were used at the following concentrations: ampicillin (100 µg/mL), kanamycin (100 µg/mL), polymyxin B (10 U/mL), and streptomycin (2,500 µg/mL). P_tac_-inducible plasmids were induced with 1 mM isopropyl β-D-1-thiogalactopyranoside (IPTG).

### Plasmid and strain construction

Plasmids and oligonucleotides used in this study are listed in Tables S2 and S3. Deletion strains were constructed using pKAS32 suicide vector (56). For deletions, 700 bp upstream and downstream homologous regions were cloned using the KpnI and SacI restriction sites in pKAS32 using Gibson assembly. The plasmids were electroporated into *E. coli* S-17 (λ Pir) strain and conjugated into *V. cholerae* strains. P_tac_-inducible overexpression plasmids were constructed by Gibson assembly of inserts amplified by PCR along with linearized pEVS143 plasmid using EcoRI and BamHI. Plasmids and oligonucleotides used in this study are listed in Tables S2 and S3. Deletion strains were constructed using pKAS32 suicide vector (56). For deletions, 700 bp upstream and downstream homologous regions were cloned using the KpnI and SacI restriction sites in pKAS32 using Gibson assembly. The plasmids were electroporated into *E. coli* S-17 (λ Pir) strain and conjugated into *V. cholerae* strains. P_tac_-inducible overexpression plasmids were constructed by Gibson assembly of inserts amplified by PCR along with linearized pEVS143 plasmid using EcoRI and BamHI. For protein expression, pET28MBP vector (57) was amplified using Phusion DNA polymerase (NEB) in GC buffer (NEB) with primers oMJF001 and oMJF002. PCR product was treated with DpnI and used as the template for a secondary amplification using primers oMJF001 and oMJF172 to add a 3′ StrepII tag to the cloning site. *zpdA* was amplified from gDNA using Q5 polymerase (NEB) with primers oMJF173 and oMJF174. Vector and insert PCR products were mixed 1:1 and transformed into chemically competent DH5α *E. coli* following the FastCloning procedure (58).

### Motility assay

For motility assays, the strains were grown in solid media containing 0.375% agar with antibiotics and IPTG as needed. Pipette tips were used to stab the plates from an overnight culture. The plates were inverted and incubated overnight at 35°C in a humidity chamber and imaged using a gel imager. The area of motility was analyzed using the Hough Circle transform package from the UCB Vision Sciences plugin in Fiji (59).

### Biofilm assay

Biofilm formation was determined using the minimum biofilm eradication concentration (MBEC) assay as described previously (60). In this assay, bacterial cells are grown on MBEC 96-well plate that contains coated pegs (Innovotech) with media. After growth, the biofilm is attached to the pegs on the lid of the plate. After washing, the attached biofilm cells are enumerated using Bac-titer Glo reagent (Promega), which measures the ATP with thermostable luciferase from viable biofilm cells. Briefly, a 1:1,000 dilution of the overnight culture was inoculated into 150 mL of media and incubated overnight at 35°C in a humidity chamber. The MBEC lid was washed with phosphate-buffered saline (PBS) for 5 minutes to remove non-adherent cells. Following washing, the MBEC lid was transferred to a 96-well black plate (PerkinElmer) containing 150 mL of 40% (vol/vol) Back-titer Glo (Promega) diluted in PBS and incubated for 10 minutes at room temperature. Following incubation, luminescence was measured using EnVision Multilabel Plate Reader (PerkinElmer).

### Promoter-lux analysis

Overnight cultures were diluted 1:1,000 in LB containing pAAR6 encoding the promoter region of *zpdA* in PBBR-lux. The cells were grown with shaking at 35°C in tubes. 150 µL of the culture was pipetted into 96-well plates at 2 hours (for low cell density) and 22 hours (for high cell density). The luminescence and OD_600_ were measured using an EnVision Multilabel Plate Reader (PerkinElmer).

### Intracellular cdG measurement

The intracellular cdG was quantified as described (61). Briefly, 1 mL of the culture was spun down, and the supernatant was removed. The cell pellets were resuspended in 100 µL cold nucleotide extraction solution (40% acetonitrile, 40% methanol, 0.1% formic acid, and 19.9% water) and incubated at −20°C for 20 minutes to release the nucleotides. The samples were centrifuged, and the supernatant was transferred to a fresh tube and dried under vacuum. The dried samples were resuspended in 100 µL HPLC-grade water and analyzed by LC-MS/MS. The concentration of cdG was determined by interpolating with known cdG standards. cdG levels were normalized to total intracellular volume to determine the molarity.

### Protein purification

For MBP-ZpdA and EDTA MBP-ZpdA protein purification, the expression plasmid was transformed into BL21(DE3) Star *E. coli* (Invitrogen). Overnight culture was used to inoculate 1 L of complete autoinduction media (62) with 100 µg/mL kanamycin (62). Cultures were shaken at 120 RPM for 6 hours at 37°C followed by 16 hours at 18°C. Cell pellets were harvested, washed with PBS, and frozen at −80°C. To purify the proteins, cell pellets were thawed in an ice-water bath and resuspended in the following lysis buffer: 25 mM HEPES KOH at pH 7.5, 5% glycerol, 300 mM NaCl, 2.5 mM 2-mercaptoethanol, 5 mM EDTA, and 0.5% CHAPS along with protease inhibitors (Complete protease inhibitor tablets, Sigma). Resuspended cell slurry was homogenized through a pre-chilled microfluidizer (Microfluidics, Newton, MA, USA). The lysate was clarified by centrifugation at 10,000 RPM in an RC5B centrifuge (Sorvall) with an SLA-1500 rotor at 4°C for 30 minutes. Clarified lysate was filtered through a 0.45 µm syringe filter (Millipore, St. Louis, MO, USA) prior to purification. Filtered lysate was loaded onto a 5 mL MBPTrap column (Cytiva, Marlborough, MA, USA) affixed to an AKTA Purifier FPLC (Cytiva) at 2 mL/minute using a sample pump in a 4°C refrigerated case. The column was washed with 20 CV of buffer consisting of 25 mM HEPES KOH at pH 7.5, 5% glycerol, 300 mM NaCl, 2.5 mM 2-mercaptoethanol, 5 mM EDTA, followed by 10 washes in 10 CV of EDTA-free buffer. Protein was eluted in column buffer supplemented with 10 mM maltose. Fractions were pooled and dialyzed overnight at 4°C against a storage buffer of 25 mM HEPES KOH at pH 7.5, 10% glycerol, 300 mM NaCl, and 5 mM 2-mercaptoethanol. Purified protein was quantified by *A*_280_ using a Nanodrop and the calculated extinction coefficient of 118,160 M^−1^ cm^−1^. Purified protein was analyzed on SDS-PAGE gels, followed by staining with Coomassie. Batches of the protein were also purified without the addition of EDTA to the lysis and column buffers. Thus, EDTA was not present in any purified protein used in subsequent enzyme activity assays.

### Protein activity assay

Phosphodiesterase activity of wild-type and EDTA-treated protein was determined as described previously with modifications (47). Protein activity was carried out in a 100 µL volume in a buffer containing 50 mM Tris HCl at pH 9.5, 50 mM NaCl, along with MnCl_2_, ZnCl_2_, MgCl_2_, CaCl_2_, and FeSO_4_ at a concentration of 1 mM. To this, 0.2 µM enzyme and 2.5 µM cdG were added and incubated at room temperature for 10 minutes. The enzyme was denatured at 90°C for 5 minutes, then cooled and mixed 1:1 with DMHA buffer (8 mM *N*,*N*-dimethylhexylamine [DMHA] + 2.8 mM acetic acid in water, pH ∼9). The pGpG product was analyzed using LC-MS/MS as described, along with the standards of known concentration of pGpG (63).

### qRT-PCR assays

Overnight cultures were sub-cultured 1:1,000 in respective media. For low cell density, OD_600_ of 0.1–0.2 and for HCD, OD_600_ of 2.0–2.5 were used. One milliliter of each replicate was pelleted, and RNA was isolated using TRIzol reagent (Thermo Fisher) according to the manufacturer’s instructions. RNA quantity was determined by NanoDrop. 5 µg of RNA was treated with Turbo DNase (Thermo Fisher), and cDNA synthesis was performed using Superscript III reverse transcriptase (Thermo Fisher). cDNA (0.78 ng) was added to 0.625 µM of each primer with 2× SYBR Green Master Mix (Applied Biosystem) in a 25 µL reaction. The reactions were performed in technical triplicates with three biological replicates for each sample, including no reverse transcriptase (no RT) control to monitor for contaminating genomic DNA. qRT-PCR conditions are 95°C for 20 seconds, then 40 cycles of 95°C for 2 seconds, and 60°C for 30 seconds in the QuantStudio 3 Real-Time PCR system (Applied Biosystems). *gyrA* was used as an endogenous reference gene for relative quantification (Δ*C*_t_). Relative fold expression was calculated using the 2^ΔΔCt^ method (64).

### ICP-MS analysis

All sample preparation steps were performed using ultrapure Milli-Q water (18.2 MΩ cm, 25°C) and metal-free polypropylene tubes (15 mL and 50 mL, Labcon). For pellet samples, overnight cultures of WT and Δ*znuABC* at 1:1,000 were inoculated into 10 mL of minimal media with or without 10 µM ZnSO_4_. The strains were grown for 5 hours, after which the cells were washed thrice with 7 mL of 240 mM sucrose solution and centrifuged at 4,000 × *g* for 10 minutes. One hundred microliters was plated to determine colony-forming units at the last wash. After centrifugation, the supernatant was removed, and the pellets were completely dried at 70°C overnight with the conical tube caps loosened. Dried bacterial samples were digested in 150 µL of trace metal-grade nitric acid (Fisher Chemical, cat. no. A509P212) at 70°C for 2–3 hours or until completely digested. After cooling for 1–2 hours at room temperature, samples were diluted with 4.85 mL of ultrapure water to achieve a final nitric acid concentration of 3%. For powder and culture media samples, the amounts were precisely weighed and subjected to the same digestion protocol described above. Throughout all ICP-MS sample processing steps, empty tubes and samples were weighed to ensure accurate sample volume determination for subsequent concentration analysis.

Elemental quantification was performed on an Agilent 8900 Triple Quadrupole ICP-MS system equipped with an SPS 4 Autosampler, integrated sample introduction system (ISIS), x-lens configuration, and micromist nebulizer. The instrument was tuned using a tuning solution (Agilent, 5185-5959). Gas mode-specific tuning was performed for both helium kinetic energy discrimination (KED) and oxygen reaction modes. An internal standardization approach was implemented using the ISIS valve system with a 200 ppb multi-element solution, containing Bi, In, ^6^Li, Sc, Tb, and Y (IV-ICPMS-71D, Inorganic Ventures). The following isotopes were monitored for comprehensive elemental analysis: ^23^Na, ^24^Mg, ^39^K,^44^Ca, ^31^P^16^O, ^32^S^16^O, ^52^Cr, ^55^Mn, ^56^Fe, ^57^Fe, ^58^Ni, ^59^Co, ^60^Ni,^63^Cu,^64^Zn, ^65^Cu, ^66^Zn. Internal standardization was achieved using ^45^Sc and ^89^Y. Calibration standards were prepared by serial dilution of a 10 ppm multi-element stock solution (IV-65024, Inorganic Ventures). A comprehensive 15-point calibration curve was established with standards from 0.02 to 800 ppb in 3% nitric acid matrix. Elemental concentrations in experimental samples were calculated by interpolation from these calibration curves using Mass Hunter software (Agilent).

## SUPPLEMENTAL MATERIAL

Supplemental Figures mbio.02275-25-s0001.pdf Fig. S1-S6.

Supplemental Tables mbio.02275-25-s0002.pdf Tables S1 and S2.

ASM does not own the copyrights to Supplemental Material that may be linked to, or accessed through, an article. The authors have granted ASM a non-exclusive, world-wide license to publish the Supplemental Material files. Please contact the corresponding author directly for reuse.
