# Supplementary material for: A cyclic di-GMP phosphodiesterase in the VSP-2 island of Vibrio cholerae is regulated by zinc and quorum sensing
Source: mBio. 2025 Sep 24;16(11):e02275-25. doi: 10.1128/mbio.02275-25 (PMC12607878; doi:10.1128/mbio.02275-25)
Supplement: Supplemental Figures — Fig. S1-S6. [file mbio.02275-25-s0001.pdf]

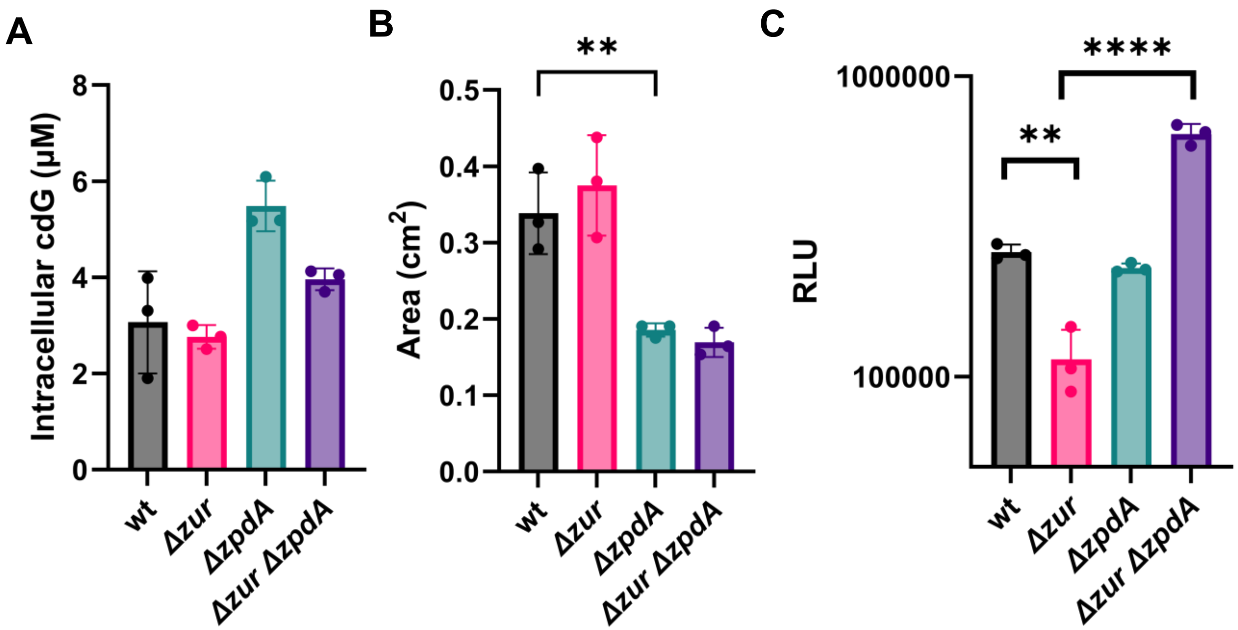

Fig. S1: (A) Intracellular cdG levels in wild-type, Δ*zur,* Δ*zpdA* and *Δzur* Δ*zpdA* for cells grown in minimal media. For comparison, wild-type and Δ*zpdA* quantifications from Figure 1F are repeated. (B) Quantification of motility by measuring the colony area on LB plates for wild-type, Δ*zur,* Δ*zpdA* and Δ*zur* Δ*zpdA* cells*.* For comparison wild-type and Δ*zpdA* quantifications from Figure 1G are repeated. (C) Quantification of biofilm expressed as relative luminescence units in wild-type, Δ*zur,* Δ*zpdA* and Δ*zur* Δ*zpdA.* Mean and standard deviation of three biological replicates is indicated. Statistical significance was determined by one-way ANOVA and post-hoc Tukey test (* = $p < 0.05$, *** = $p < 0.005$, **** = $p < 0.0005$).

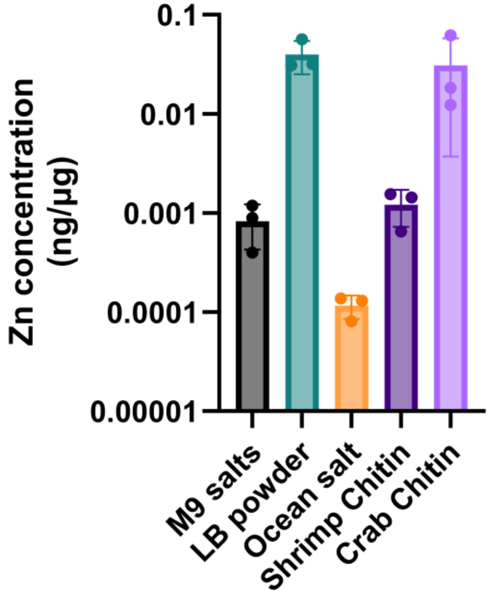

Fig. S2: Inductively Coupled Plasma Mass Spectrometry (ICP-MS) quantification of Zn content of growth media: M9-minimal salt, LB powder, ocean salt, shrimp and crab chitin. The average of three independent replicates is indicated.

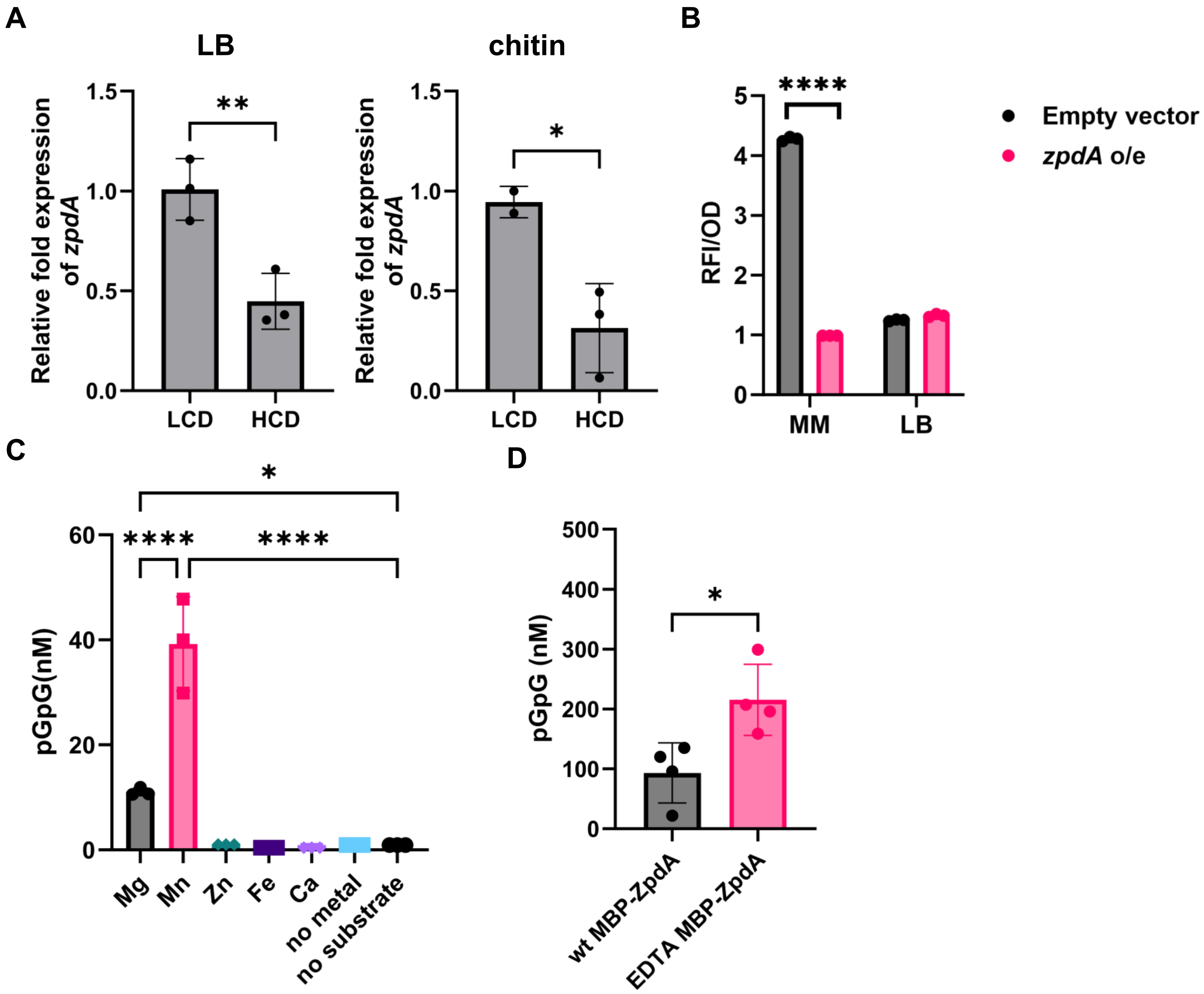
A
LB
chitin
Relative fold expression of zpdA
1.5
1.0
0.5
0.0
**
*
LCD
HCD
B
5
4
3
2
1
0
RFI/OD
****
MM
LB
Empty vector
zpdA o/e
C
pGpG(nM)
60
40
20
0
*
****
****
Mg
Mn
Zn
Fe
Ca
no metal
no substrate
D
pGpG (nM)
500
400
300
200
100
0
*
wt MBP-ZpdA
EDTA MBP-ZpdA

Fig. S3: (A) Relative expression of *zpdA* in E7646 strain at low cell density (LCD) (OD_{600} – 0.1) and high cell density (HCD) (OD_{600} 1.5-2) in LB and chitin media. Mean and standard deviation of three biological replicates is indicated. Statistical significance was determined by t-test (* = $p < 0.05$, *** = $p < 0.005$, **** = $p < 0.0005$). (B) cdG binding RFP biosensor readout normalized to OD_{600} from overexpression of *zpdA* and empty vector in minimal media and LB. For comparison, *zpdA* overexpression and empty vector in minimal media values from Figure 1B. Mean and standard deviation of three biological replicates is indicated. Statistical significance was determined by t-test (* = $p < 0.05$, *** = $p < 0.005$, **** = $p < 0.0005$). (C) Concentration of pGpG measured by LC-MS/MS in wild-type and EDTA-treated protein with cdG substrate in the presence of 1 mM $MgCl_2$, 1 mM $MnCl_2$, 1 mM $ZnCl_2$, 1 mM $FeSO_4$, 1 mM $CaCl_2$, along with no metal and no substrate controls. The mean and standard deviation of three replicates are indicated. (D) Concentration of pGpG measured by LC-MS/MS in wild-type and EDTA-treated protein with cdG substrate in the presence of 1 mM $MnCl_2$.Mean and standard deviation of 4 replicates is indicated. Statistical significance was determined by one-way ANOVA and post-hoc Tukey test (* = $p < 0.05$, *** = $p < 0.005$, **** = $p < 0.0005$).

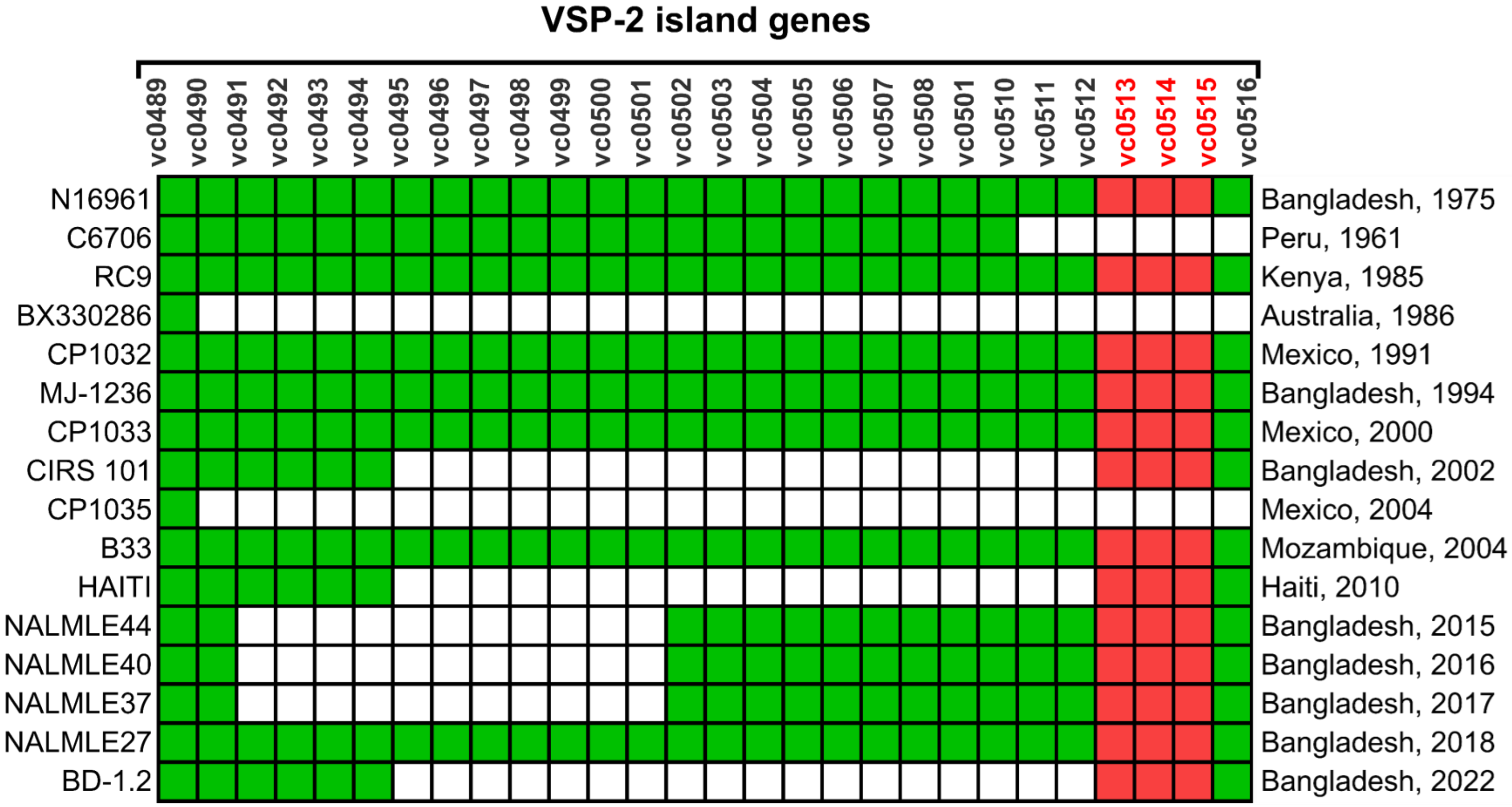

Fig. S4: Comparison of VSP-2 island genes of different El Tor strains are shown. *vc0513 (verA), vc0514, vc0515 (zpdA)* genes are represented in red, other genes are represented in green. Filled and unfilled box represents the presence of gene and absence of gene respectively.

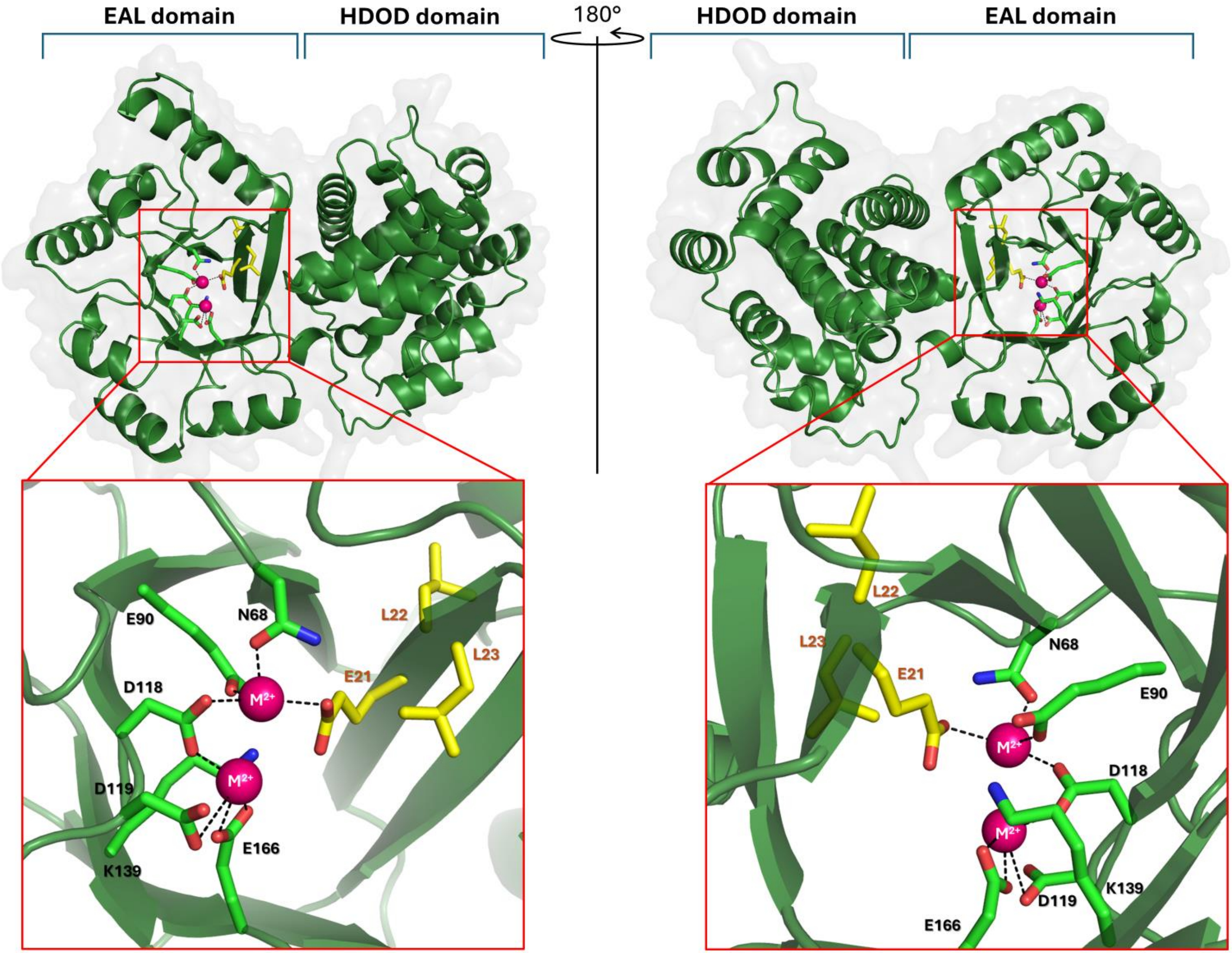
EAL domain
HDOD domain
180°
HDOD domain
EAL domain
E90
N68
L22
L23
E21
D118
M2+
M2+
D119
E166
K139
L22
L23
E21
N68
E90
M2+
D118
M2+
K139
D119
E166

Fig. S5: AlphaFold-3 predicted structure of ZpdA protein (AF-Q9KUK3-F1-v4) showing the metal-binding site and domain organization. The structure is displayed highlighting the N-terminal EAL domain and C-terminal HDOD domain arrangement, with two M^{2+} ion (indicates any divalent cation which is predicted to bind to the same site) binding simulated through the AlphaFold Server. Red boxes indicate magnified views of the proposed metal binding site. Conserved residues (E-N-E-E-D-K-E) determined to be involved in metal binding in other EAL proteins are indicated (E90, N68, D118, D119, E166, and K139 shown as green sticks) in proximity to two metal ions (purple spheres), with black dashed lines indicating atoms within 3.0Å distance. The ELL motif residues (E21, L22 and L23, shown in yellow sticks) are positioned near the metal-binding site, with E21 participating in metal coordination.

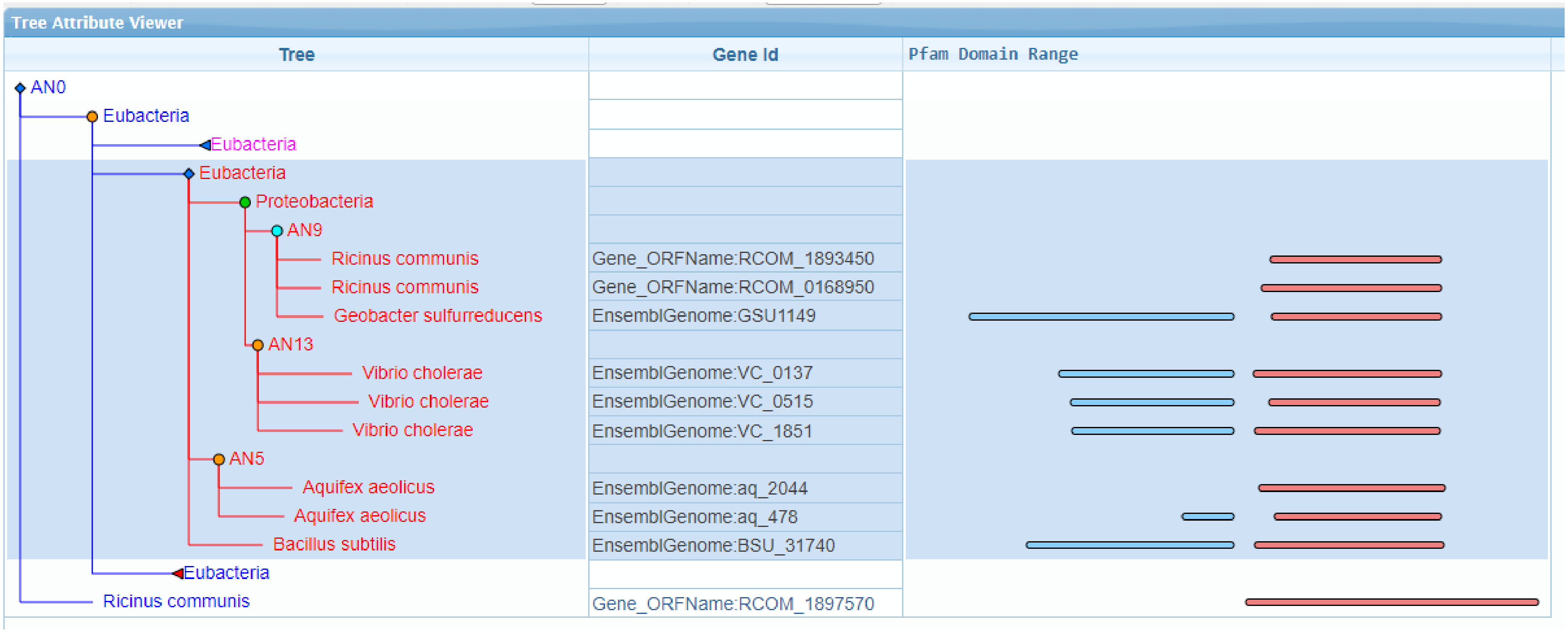

Fig. S6: PANTHER tree viewer (Protein Analysis Through Evolutionary Relationships) of ZpdA protein. Orange circles represent duplications, blue circles represents horizontal gene transfers, green circles represents speciation node, and blue square represents expanded subfamily change node. Blue bars represents EAL domains and pink bars represents HDOD domains of Pfam domain classification.
