# Supplementary material for: A cyclic di-GMP phosphodiesterase in the VSP-2 island of Vibrio cholerae is regulated by zinc and quorum sensing
Source: mBio. 2025 Sep 24;16(11):e02275-25. doi: 10.1128/mbio.02275-25 (PMC12607878; doi:10.1128/mbio.02275-25)
Supplement: Supplemental Tables — Tables S1 and S2. [file mbio.02275-25-s0002.pdf]

Supplementary tables

Table S1- List of strains used in this study

Strains	Description	Reference
V. cholerae		
N16961 El Tor		Obtained from Tobias Dörr lab
SGM-110	N16961 Δ*zur*	[1] (Obtained from Tobias Dörr lab)
N16961 *Δvc0515*	N16961 Δ*zpdA*	Obtained from Tobias Dörr lab
SGM-112	N16961 Δ*znuABC*	[2] (Obtained from Tobias Dörr lab)
AAR077	*N16961Δzur ΔzpdA*	This study
E7946 El Tor		Obtained from Kim Seed Lab
CW2034	C6706 Δ*vpsL*	[3]
CW2036	CW2034 Δ*hapR*	[3]
CW2037	CW2034 Δ*luxO*	[3]
AAR156	N16961 Δ*vpsL*	This study
E. coli		
S17-λpir	Tpr Smr recA thi pro hsdR17(rK-K+) RP4::2-Tc::Mu Km Tn7 λpir	Lab stock

Table S2- List of plasmids used in this study

Plasmid	Description	Reference
pKAS32	λpir dependent suicide vector, AmpR	[4]
pEVS143	*pTac* overexpression vector, KanR	[5]
pBBR-lux	promoterless pBBR1 plasmid with lux genes of *V. harveyi*, CamR	[6]
pSLS13	*hapR* cloned in pEVS143, KanR	[7]
pEVS141	Promoterless pEVS143 vector control, KanR	[8]
pVC1353	*vc1353* in pEVS143, KanR	[9]
pCMW121	*vc1086* in pEVS143, KanR	[3]
pRP0122_*Pbe_amcyan_Bc3-4_turborfp*	cdG biosensor plasmid, AmpR	[10]
pLJW01	*zpdA* in pEVS143, KanR	This study

pET28-MBP-ST	vector for protein expression, KanR	[11]
pAAR14	*zpdA*^{AAA} in pEVS143, KanR	This study
pAAR6	P*vc0515* in pBBR-lux, CamR	This study
pAAR19	*zpdA* in in pET28-MBP-ST, KanR	This study

Table S3- List of oligonucleotides used in this study

Primer	Sequence	Description	Reference
AAR049	ggatatctatttttctcactattcctaaacgccgccgcataaccaacgatagttaagttctcatctg, FOR	ELL-AAA mutation in *zpdA*	This study
AAR050	cagatgagaacttaactatcgttggttatgcggcggcgtttaggaatagtgagaaaaatagatatcc, REV	ELL-AAA mutation in *zpdA*	This study
AAR093	aacgttaacaaccggtacctGAATTCTCTGAGTTAGCATTGGG, FOR	upstream region of *zpdA* for cloning in pKAS32	This study
AAR094	aaagactataGCAGCACCACCGCTATTAAC, REV	upstream region of *zpdA* for cloning in pKAS32	This study
AAR095	gtggtgctgcTATAGTCTTTGGTTAAATTTATCAC, REV	downstream region of *zpdA* for cloning in pKAS32	This study
AAR096	gcgctttaaatttgcgcatgTGGTAACTATCGAACCAG, REV	downstream region of *zpdA* for cloning in pKAS32	This study
AAR140	cggtggcggccgctctagaaGGCGTTTTGTTGATTCAAGATTAG	cloning Pvc0515 in pMMBlux	This study
AAR141	ttttgcggccgcaactagagGCAGCACCACCGCTATTAAC	cloning Pvc0515 in pMMBlux	This study
AAR160a	TGCTAATAGATGGCTACGCGG, FOR	qPCR primer for amplifying *zpdA*	This study
AAR160b	TCCCCGAATAACGGAAAGCA, REV	qPCR primer for amplifying *zpdA*	This study
CMW2926	TGGCCAGCCAGAGATCAAG, FOR	qPCR primer for amplyfying *gyrA*	[12]
CMW2927	ACCCGCAGCGGTACGA, REV	qPCR primer for amplifying *gyrA*	[12]
oMJF001	tccttgaaaataaagattttcGGATCCGGATTGGAAGTACAGG	Amplify pET28MBP-ST Vector	This study
oMJF002	TGAGATCCGGCTGCTAACAAAG	Amplify pET28MBP Vector	This study

oMJF172	GGTTGGAGCCACCCGCAGTTCGAAAAATAGTGAGATCC	Amplify pET28MBP-ST Vector	This study
oMJF173	atctttattttcaaggaATGGATAGTTTCATAGCAAAACAGG	Cloning *zpdA* into pET28MBP	This study
oMJF174	tgcgggtggctccaaccTTTAACTTCTTTCATTAAAGTTCGACACTC	Cloning *zpdA* into pET28MBP-ST	This study

References:

1. Murphy, S. G. *et al.* Endopeptidase Regulation as a Novel Function of the Zur-Dependent Zinc Starvation Response. **10**, 15 (2019).
2. Murphy, S. G., Johnson, B. A., Ledoux, C. M. & Dörr, T. Vibrio cholerae's mysterious Seventh Pandemic island (VSP-II) encodes novel Zur-regulated zinc starvation genes involved in chemotaxis and cell congregation. *PLoS Genet* **17**, e1009624 (2021).
3. Waters, C. M., Lu, W., Rabinowitz, J. D. & Bassler, B. L. Quorum Sensing Controls Biofilm Formation in *Vibrio cholerae* through Modulation of Cyclic Di-GMP Levels and Repression of *vpsT*. *J Bacteriol* **190**, 2527–2536 (2008).
4. Skorupski, K. & Taylor, R. K. Positive selection vectors for allelic exchange. *Gene* **169**, 47–52 (1996).
5. Bose, J. L., Rosenberg, C. S. & Stabb, E. V. Effects of *luxCDABEG* induction in *Vibrio fischeri:* enhancement of symbiotic colonization and conditional attenuation of growth in culture. *Arch Microbiol* **190**, 169–183 (2008).
6. Lenz, D. H. *et al.* The Small RNA Chaperone Hfq and Multiple Small RNAs Control Quorum Sensing in *Vibrio harveyi* and *Vibrio cholerae*. *Cell* **118**, 69–82 (2004).
7. Svenningsen, S. L., Waters, C. M. & Bassler, B. L. A negative feedback loop involving small RNAs accelerates *Vibrio cholerae* 's transition out of quorum-sensing mode. *Genes Dev.* **22**, 226–238 (2008).
8. Dunn, A. K., Millikan, D. S., Adin, D. M., Bose, J. L. & Stabb, E. V. New *rfp* - and pES213-Derived Tools for Analyzing Symbiotic *Vibrio fischeri* Reveal Patterns of Infection and *lux* Expression In Situ. *Appl Environ Microbiol* **72**, 802–810 (2006).
9. Massie, J. P. *et al.* Quantification of high-specificity cyclic diguanylate signaling. *Proc. Natl. Acad. Sci. U.S.A.* **109**, 12746–12751 (2012).
10. Zhou, H. *et al.* Characterization of a natural triple-tandem c-di-GMP riboswitch and application of the riboswitch-based dual-fluorescence reporter. *Sci Rep* **6**, 20871 (2016).
11. Lee, S. W. *et al.* Discovery of a widely distributed toxin biosynthetic gene cluster. *Proc. Natl. Acad. Sci. U.S.A.* **105**, 5879–5884 (2008).
12. Hsueh, B. Y. *et al.* Phage defence by deaminase-mediated depletion of deoxynucleotides in bacteria. *Nat Microbiol* **7**, 1210–1220 (2022).
